# Evaluation of Change in Microhardness by Application of MI Varnish on Primary Tooth Enamel, Affected by Use of Frequently Prescribed Paediatric Syrups: An In Vitro Study

**DOI:** 10.7759/cureus.6533

**Published:** 2020-01-01

**Authors:** Ankita Maurya, N.D. Shashikiran, Namrata Gaonkar, Sachin Gugawad, Swapnil Taur, Savita Hadakar, Pradnya Chaudhari

**Affiliations:** 1 Department of Paedodontics and Preventive Dentistry, School of Dental Sciences, Krishna Institute of Medical Sciences, Karad, IND

**Keywords:** erosion, microhardness, syrups

## Abstract

Introduction

Dental erosion is considered as an irreversible progressive loss of tooth structure due to chemical dissolution by acids not of bacterial origin. Frequent intake of syrups can be an effective factor in tooth erosion when prescribed for illnesses. In the acidic environment, MI Varnish releases calcium, phosphate and fluoride from the covering layer and the teeth can be prevented from such acidic attacks from the erosive content of the syrups.

Aim

To evaluate change in microhardness by application of MI Varnish on primary tooth enamel, affected by use of frequently prescribed paediatric syrups.

Method

The effects of three paediatric syrups (Ibugesic Plus, Novamox, Becozinc H) and distilled water (control group) with different ingredients on primary tooth enamel were evaluated. Immersion cycles were applied three times a day for one minute. The measurements of the samples prepared were taken on 0 (baseline), third, fifthand seventh day. Microhardness was measured using a Vickers hardness tester. Then, those samples were coated with MI Varnish, and changes in microhardness were evaluated using the Vickers hardness tester after one week (14th day). The pH, titratable acidity and buffer capacity of the syrups were also evaluated.

Results

There was a significant decrease in microhardness in all the syrups (p < 0.001) on days 3, 5, 7 and 14. Novamox syrup and distilled water (control group) were the least erosive on the primary tooth enamel and Becozinc H syrup being the most erosive on primary tooth enamel was seen. MI Varnish remineralized the enamel but not with any significant difference.

Conclusion

In our findings, Novamox syrup showed the least erosion on primary tooth enamel compare to other syrups. Enamel microhardness was significantly reduced on all days after immersion in all syrups. Application of MI Varnish to enamel resisted erosion but was not significant. Paedodontists should be aware of the erosive potential of all the frequently prescribed syrups and stress on compliance with oral hygiene measures and application of varnishes releasing fluoride, calcium and phosphate on the tooth.

## Introduction

Dental erosion is defined as an irreversible progressive loss of hard dental tissues by chemical dissolution without bacterial involvement [1]. The erosion begins with the softening of the enamel surface, mainly characterized by a reduction in microhardness [2]. Intrinsic aetiological factors are caused by the contact of dental tissue with stomach acids which include eating disorders, regurgitation and reflux. External aetiological factors include acidic drugs, diet, environment and behavioural factors. Epidemiological data collected during in vitro and in situ studies have shown that erosion, one of the three toothwear processes, is the most common cause of tooth surface loss. Erosion was first included in the dental health surveys of children living in England in 1993 and since that time has been periodically surveyed [3].

Liquid paediatric medicines are part of the daily routine of children with chronic illnesses. Frequent intake of drugs with erosive potential can be an effective factor in tooth erosion. Liquid oral medications are usually prescribed to ensure compliance with drug intake in children. Acidic preparations are considered necessary for drug distribution, chemical stability, physiological adaptation and flavour enhancement. There is paucity in literature about evaluation of enamel microhardness, after paediatric syrup usage and remineralizing potential of MI Varnish on it. Hence, this study was carried out to evaluate the effects of frequently prescribed paediatric syrups on the primary tooth enamel microhardness in vitro.

## Materials and methods

This is an in vitro study conducted during the period of two months. In this study, four paediatric syrups with different ingredients were used, i.e., Ibugesic Plus syrup, Novamox syrup, Becozinc H syrup and the control group consisted of teeth immersed in distilled water. The drugs used were selected among long-term and commonly used syrups that are frequently prescribed by paedodontists for acute or chronic diseases.

Sample size determination for minimum number of teeth required for each syrup was done by using the below formula:  ​​​​​​ 

n= ​​​( z_alpha _+ z_beta _)^2 ^* ( s_1_^2^ + s_2_^2 ^) / ( m_2_-m_1 _)^2^

where z_alpha_ = 1.96 type I error at 95% level of significance and z_beta_ = 1.037 type II error with 85% power.

The values of m_1 _= 243.3, m_2_ = 335.1 , s_1_ = 50.3 and s_2 _= 36 [4].

Therefore, the minimum number of teeth required is 4 for each syrup according to the values obtained from the formula. Hence, the total sample size equals to 16 primary single rooted teeth.

In this study, 16 non-cariogenic, primary single rooted teeth that were freshly extracted were included. Teeth with incisor hypoplasia, decayed and with white spot lesions were excluded from the study.

Once the teeth were collected, they were kept in 0.5% chloramine T solution. Prior to use, the teeth were cleaned with scaler tip for removing the debris and polished with prophylaxis paste using a polishing brush with a low-speed handpiece.

While the samples were being prepared, the teeth were separated from their roots by a transverse section through the cementoenamel junction (CEJ) with the help of a water-cooled diamond saw with a rapidly rotating handpiece. Subsequently, each crown was embedded in the acrylic resin with the incisal surface of the crown facing downwards inside the resin and the CEJ surfacing facing upwards.

After the resin acrylic polymerization of each tooth, the CEJ surfaces of the samples were flattened with 600, 900 and 1,200 grit aluminium oxide (Al_2_O_3_) abrasive papers for non-slip measurements and to obtain a flat, stable surface without the dentin coming out. The samples were ultrasonically cleaned in deionized water for 10 minutes.

The microhardness values of the initial enamel surfaces (baseline values) were evaluated using a Vickers hardness tester. A 50 g of load was applied through the indenter with a dwell time of 12 seconds. Three readings were taken for each specimen, and the mean of the measurements was calculated as the Vickers hardness number. The testing was done on the outer surface of enamel, i.e., on the edge of tooth sample.

For the first parameter, i.e., pH measurement, titratable acidity (TA) and buffering capacity (BC), the pH value of the syrups used for the immersion cycles and the amount of base (acid-base titration) required to raise pH to 7.0 were measured with a digital pH meter. The amount of 10 ml of each paediatric syrup in a glass beaker was placed, and a glass electrode was inserted into the syrup displaying pH on the meter. Each syrup was tested three times to record a mean measurement. To measure the TA, 10 g of each solution was titrated with 0.5 M NaOH in 0.1-ml increments at 25°C. The BC was calculated with the following equation:

β = −ΔC/ΔpH

where β = buffering capacity, ΔC = the amount of base used, ΔpH = the change in pH resulting from base addition [4].

For the second parameter, after the baseline microhardness measurements, 16 acrylic blocks were assigned randomly to four separate groups for the samples to be immersed in the syrups; one of them was assigned to distilled water (control group). During the immersion cycles, the samples were submerged for one minute every eight hours, three times a day, in 5 ml of an undiluted drug solution. During each immersion cycle, the solutions were shaken prior to the immersion to ensure the homogeneity of the drug solutions. After each immersion cycle, the samples were washed with distilled water, and until the next immersion cycle, they were kept in 10 ml of saliva at a temperature of 37°C.

This procedure was repeated for one week, and a total of 21 immersion cycles were performed. The solutions and saliva were changed daily for each sample. In the control group, the distilled water was changed daily. The surface microhardness was tested at third, fifth and seventh day after continuous and systematic repetition of the daily immersion cycles. The samples were kept in distilled water during the days they were tested.

After the immersion cycles were completed on the seventh day, a thin layer of MI Varnish application was done using a microbrush on the enamel surfaces of the samples for one minute and then was stored in saliva until the testing was done. After one week, i.e., on 14th day, change in the microhardness was evaluated using the Vickers hardness tester.

Repeated measures analysis of variance (ANOVA) was done to check the change in microhardness on all days (day 0 to 14). Statistical analysis was conducted using InStat software (GraphPad, San Diego, CA). One-way ANOVA was done to check change in microhardness in between all four syrups.

## Results

pH measurement, TA and BC results: The pH values ranged from 3.6 (Becozinc H syrup) to 5.56 (Novamox syrup). Ibugesic Plus had the highest TA (3.71 g/100 ml), whereas Becozinc had the lowest TA (1.43 g/100 ml). Additionally, Ibugesic Plus had the highest BC (1.33 g/100 ml), whereas Becozinc had the lowest BC (0.42 g/100 ml) (Table 1).

**Table 1:** The pH, titratable acidity (TA) and buffering capacity (BC) values of the syrups with the volume of NaOH (VNaOH)

Pharmaceutical name	pH	V_NaOH _	Titratable acidity	Buffering capacity
Ibugesic Plus	4.22	3.6 ml	3.71 g/100ml	1.33 g/100ml
Novamox syrup	5.56	0.9 ml	1.88 g/100ml	1.3 g/100ml
Becozinc H syrup	3.6	1.3 ml	l.43 g/100 ml	0.42 g/ml
Distilled water	5.49	-	-	-

Surface microhardness results: The measurements of the tooth samples, which were immersed in the Ibugesic Plus syrup, Novamox syrup, Becozinc H syrup and distilled water demonstrated a significant difference between days 0, 3, 5, 7 and 14 of the study (p < 0.001) (Table 2). 

**Table 2:** Measurement of the microhardness of the tooth samples immersed in the drug solutions on days 0, 3, 5, 7 and 14 Data presented as mean±standard deviation. VHN, Vickers Hardness Number

Pharmaceutical name	Day 0 (baseline) VHN score	Day 3 VHN score	Day 5 VHN score	Day 7 VHN score	Day 14 VHN score	p value
Ibugesic Plus	94.8±3.5	85.9±4.5	85.6±2.9	82.5±4.5	80.9±4.9	<0.001
Novamox syrup	104.6±3.9	118.5±6.7	104.3±5.4	101.6±1.9	100.4±1.8	<0.001
Becozinc H syrup	87.1±0.9	73.3±2.4	68.9±1.8	63.7±1.8	62.7±1.02	<0.001
Distilled water	99.2±2.9	99.9±2.9	97.4±3.9	98.5±3.9	96.4±4.8	0.4
p value	<0.001	<0.001	<0.001	<0.001	<0.001	

## Discussion

According to Yilmaz et al., a drug with a low pH value and high TA and BC has the potential to produce abrasive lesions in the teeth and if given to children regularly or for a long time have the erosive potential [4].^ ^Therefore, the analysis of their pH is an important factor while studying dental erosion.

The pH values ranged from 3.6 (Becozinc H syrup) to 5.56 (Novamox syrup) in this study. The range is similar to the findings of Mahmoud and Omar, where the pH of the studied medications ranged from 3.47 to 6.92 [5]. This study is also in agreement with other studies given by Cavalcanti et al. and Passos et al., where the pH ranged from 2.5 to 6.9 [6,7].

TA represents the total content of acids and is considered to determine the strength of the erosive potential of the syrup; BC is the time needed to neutralize the acid in the syrup by the saliva [4].

In this study, Ibugesic Plus had the highest TA and BC and also low pH, which makes it erosive for the enamel. This decrease can be linked to the inactive ingredients of the drug such as a sorbitol solution, orange flavour or sucrose [4]. Becozinc H syrup had the lowest TA and BC values but it also has the lowest pH so overall even that makes it erosive for the tooth. Novamox syrup had the highest pH among all which makes it less acidic among all and also had balanced TA and BC values which makes it least erosive among all. Distilled water also had an optimum pH.

Mittal et al. have concluded that majority of the paediatric liquid medicaments used in the study were acidic in nature with pH below 7 and those paediatric liquid medicaments frequently used had erosive effect on enamel of deciduous teeth [8].

In the current study, it was observed that all of the syrups used caused a significant decrease in the microhardness on all the days with a p value of <0.001 and also there is a difference in the values of microhardness in all the syrups on the same days also. This is in agreement with a study done by Kulkarni et al., where they have concluded that Ferium XT, Crocin syrup, and Ambrolite-D had a significant and gradual loss of surface microhardness on all days (7, 14, 21 and 28 days) [9].

Another study done by Yilmaz et al. stated that there was a significant difference in the surface microhardness between days 0, 7 and 14 [4].

In the current study, MI Varnish tried to remineralize all the samples but the major remineralization was done in Novamox syrup samples followed by Becozinc H syrup samples and Ibugesic Plus syrup samples. In Becozinc H syrup samples, there was a gradual decrease of microhardness from day 0 to 14, whereas in Novamox syrup samples there was less decrease in microhardness on all days compared to Becozinc H. Hence, it can be concluded in this study that Novamox syrup had the least erosive potential.

Bayrak et al. have concluded that MI varnish was most effective in increasing the enamel resistance to erosion [10].

Tuloglu et al. have concluded that MI varnish was more effective in increasing the acid resistance of primary enamel than Clinpro White and Duraphat. However, further clinical research is needed to confirm these in vitro results [11].

In this study, MI Varnish tried to remineralize the enamel to some extent but not with any significant difference.

Limitations of the study: The sample size is small, and various other class of syrups have not been included in the study for complete erosive review of all classes of paediatric syrups.

## Conclusions

In our observations, all syrups showed erosive potential but Novamox syrup showed the least erosion on primary tooth enamel. Enamel microhardness was significantly reduced on all days after immersion in all syrups. Application of MI Varnish to enamel increased its resistance to erosion but was not significant. Further studies should be carried to substantiate our findings.
